# Differential FSH Glycosylation Modulates FSHR Oligomerization and Subsequent cAMP Signaling

**DOI:** 10.3389/fendo.2021.765727

**Published:** 2021-12-03

**Authors:** Uchechukwu T. Agwuegbo, Emily Colley, Anthony P. Albert, Viktor Y. Butnev, George R. Bousfield, Kim C. Jonas

**Affiliations:** ^1^ School of Life Course and Population Sciences, Department of Women and Children’s Health, Guy’s Campus, King’s College London, London, United Kingdom; ^2^ Institute of Reproductive and Developmental Biology, Imperial College London, London, United Kingdom; ^3^ Vascular Biology Research Centre, Molecular & Clinical Science Research Centre, St George’s University of London, London, United Kingdom; ^4^ Department of Biological Sciences, Wichita State University, Wichita, KS, United States

**Keywords:** follicle-stimulating hormone receptor, follicle-stimulating hormone, gonadotropic hormones, G protein-coupled receptors (GPCR), oligomers, oligomerization

## Abstract

Follicle-stimulating hormone (FSH) and its target G protein-coupled receptor (FSHR) are essential for reproduction. Recent studies have established that the hypo-glycosylated pituitary FSH glycoform (FSH21/18), is more bioactive *in vitro* and *in vivo* than the fully-glycosylated variant (FSH24). FSH21/18 predominates in women of reproductive prime and FSH24 in peri-post-menopausal women, suggesting distinct functional roles of these FSH glycoforms. The aim of this study was to determine if differential FSH glycosylation modulated FSHR oligomerization and resulting impact on cAMP signaling. Using a modified super-resolution imaging technique (PD-PALM) to assess FSHR complexes in HEK293 cells expressing FSHR, we observed time and concentration-dependent modulation of FSHR oligomerization by FSH glycoforms. High eFSH and FSH21/18 concentrations rapidly dissociated FSHR oligomers into monomers, whereas FSH24 displayed slower kinetics. The FSHR β-arrestin biased agonist, truncated eLHβ (Δ121-149) combined with asparagine56-deglycosylated eLHα (dg-eLHt), increased FSHR homomerization. In contrast, low FSH21/18 and FSH24 concentrations promoted FSHR association into oligomers. Dissociation of FSHR oligomers correlated with time points where higher cAMP production was observed. Taken together, these data suggest that FSH glycosylation may modulate the kinetics and amplitude of cAMP production, in part, by forming distinct FSHR complexes, highlighting potential avenues for novel therapeutic targeting of the FSHR to improve IVF outcomes.

## Introduction

The actions of follicle-stimulating hormone (FSH) and its receptor (FSHR) are essential for reproduction (1–5). With critical roles in follicle maturation, recruitment, and dominant follicle selection, FSH/FSHR are pivotal for granulosa cell (GC) proliferation and estradiol production (6, 7). Consequently, FSHR is a key therapeutic target of assisted reproductive technologies (ART), where supraphysiological concentrations of recombinant and urinary FSH preparations are utilized during the ovarian stimulation phase of *in vitro* fertilization (IVF) to facilitate the recruitment and maturation of multiple antral follicles (8–10). Despite technological advances in IVF, there has been little change in the success rates (11), which are highest in women <35 and decline thereafter, highlighting the need for novel therapeutic FSH/FSHR targeting strategies to advance IVF success rates.

FSH is a complex heterodimeric glycoprotein hormone, comprised of an alpha subunit, that is common to other glycoprotein hormone family members such as thyroid-stimulating hormone (TSH), luteinizing hormone (LH) and human chorionic gonadotropin hormone (hCG), along with a hormone specific beta subunit (FSHβ). FSHβ differs in amino acid sequence and glycosylation pattern to other glycoprotein hormone beta subunits, conferring biological specificity and selectivity of FSH to FSHR (12). FSH possesses 4 Asn-linked glycosylation sites, with two sites on the alpha subunit (Asn52 and Asn78) and two sites on the beta subunit (Asn7 and Asn24). Naturally occurring differences in the macro-glycosylation pattern of FSH have been previously reported in human pituitary extracts (13), with modification of the FSHβ subunit glycosylation pattern resulting in the identification of three FSH glycoforms; partially glycosylated FSH (FSH21/18, as purified preparations possess both variants) with a single glycosylated site at either Asn7 (FSH21) or Asn24 (FSH18) and fully glycosylated FSH (FSH24) with both FSHβ subunit Asn residues glycosylated. Interestingly, age-dependent differences in pituitary expression levels of FSH21/18 and FSH24 have been reported. FSH21/18 has been shown to be predominant in pituitary extracts from women in their 20’s, decreasing thereafter, with a concomitant increase in FSH24 expression, resulting in FSH24 predominating in menopausal-aged women (13). Functional analysis has shown that this difference in glycosylation pattern results in modulation of binding to the FSHR (14) and the amplitude of the canonical FSHR signaling pathway, the Gαs/cAMP/PKA signaling, with FSH21/18 displaying faster binding kinetics and more potent activation of Gαs signaling (15–17). Recent studies suggest that FSH glycoforms may display distinct signaling profiles, or signal bias (18) in activation of AKT (19, 20), β-arrestin (17) and calcium signaling (17, 21). Important functional differences have also been observed, with differences in ovarian and testicular gene expression in mice injected with either FSH21/18 or FSH24 (19). However, how these differences in the signaling properties of FSH21/18 and FSH24 are interpreted by FSHR remain unknown.

FSHR is a Class A G protein-coupled receptor (GPCR). An increasingly important way that GPCRs have been shown to regulate ligand specificity and signal amplitude is *via* association and formation of dimers/oligomers (22–25). FSHR has been demonstrated to self-associate and homomerize (26–30), which is thought to underpin the inherent negative cooperativity displayed by FSHR (26). The FSHR has also been shown to heteromerize with the LHR (30–32) and membrane-bound estrogen receptor (GPER) (33), resulting in modulation of signal selectivity (31, 32). However, the functional role of FSHR homomerization remains to be demonstrated, and how different FSH ligands may impact FSHR homomerization to mediate their differences in signal specificity and selectivity remains unknown.

With advances in single molecule imaging technology, we are now in the position to investigate the molecular mechanisms underpinning how FSH glycoforms specify the differences observed in the kinetics and amplitude of cAMP signaling, with single molecule precision and at physiological levels of receptor density. Using a combination of the single molecule imaging technique, photoactivated dye-localization microscopy (PD-PALM) (34, 35), and differential FSH glycosylation variants- FSH21/18, FSH24, a potent FSHR stimulator- equine FSH (eFSH) and a FSHR β-arrestin biased agonist with diminished ability to activate cAMP - truncated eLHβ (Δ121-149) combined with asparagine56-deglycosylated eLHα, designated dg-eLHt (36), we have determined that FSH glycoforms differentially modulate FSHR oligomerization in both a temporal and concentration-dependent manner. These differences observed in FSHR oligomerization correlated with temporal and magnitude differences observed in cAMP production and cre-luciferase activity. These data suggest a novel mechanism by which different FSH ligands may modulate the magnitude of cAMP signal through differential regulation of FSHR oligomerization.

## Material and Methods

### Materials

Purified pituitary FSH21/18 and FSH24 (17), equine FSH (eFSH) and dg-eLHt (37) were kindly supplied by Professor George Bousfield, Wichita State University, Wichita, KS, USA. CAGE 552 fluorophore dye was purchased from Abberior. N-terminally hemagglutinin-tagged FSHR (HA-FSHR) encoded plasmid DNA were constructed as previously described (32). Primary antibody HA.11 was purchased from BioLegend^®^. Plasmid DNA encoding GloSensor™-20F, cAMP-response element-luciferase reporter gene (cre-luciferase), *Renilla* luciferase reporter gene (*R*-luciferase), GloSensor™ reagent stock and Dual-Luciferase^®^ Reporter Assay System were purchased from Promega.

### Cell Culture and Transient Transfections

HEK293 cells were maintained and cultured in 5% CO_2_ in air at 37°C in Dulbecco′s Modified Eagle′s Medium (DMEM-6429, Sigma-Aldrich) supplemented with 10% Fetal Bovine Serum (FBS-F9665, Sigma-Aldrich), and 1% Antibiotic-Antimycotic (15240062, ThermoFisher). For PD-PALM experiments, cells were transiently transfected with 3 μg HA-FSHR, and then re-plated 24 hours later onto 8-well 1.5 glass-bottomed chamber slides. For cAMP GloSensor™ and cre-luciferase activity assays, HEK293 cells were co-transiently co-transfected with 3 μg of HA-FSHR and either 1 µg GloSensor™-20F DNA plasmid or 800 ng cre-luciferase and 150 ng *R*-luciferase DNA plasmids, and cells re-plated the following day onto white clear-bottomed 96-well plates (50,000 cells/well). All transient transfections were carried out in tissue culture-treated 6-well plates (600,000 cells/well) using Lipofectamine 2000^®^ (Invitrogen) as per manufacturer’s instructions. Cells were assayed 48 hours post-transfection.

### PD-PALM

To assess FSHR monomer, dimer and oligomer populations at the plasma membrane, PD-PALM experiments were performed as previously described (32, 34, 35). Briefly, HEK293 cells expressing HA-FSHR were pre-incubated for 30 minutes with CAGE 552-labeblled HA.11 antibody at 37°C, protected from light. HA.11 primary antibodies were labeled with CAGE 552 photoswitchable dyes at a 1:1 ratio, as previously described (34, 35) and following manufacturer’s protocols (Abberior). At the end of the pre-incubation, antibodies were removed, and cells were treated with 0 (control), or either 30 or 1 ng/ml of eFSH, FSH21/18, FSH24 or dg-eLHt for 0, 2-, 5- or 15 minutes. Cells were washed with PBS and fixed for 30 minutes with 0.2% glutaraldehyde in 4% PFA at room temperature. Following fixation, cells were subsequently washed, stored and imaged in PBS using an inverted Zeiss Elyra PS.1 microscope with a 100x 1.45 NA objective lens in TIRF-mode at a rate of 10 frames/second over a total of 31,500 frames.

### Localization Analysis

Individual FSHR molecules were resolved as previously described (32, 34, 35). To summarize, cropped non-overlapping 5 x 5 μm areas, within cell boundaries, of fluorescent intensity image frames were analyzed using the QuickPALM Fiji plug-in, generating x-y coordinates of each FSHR molecule. The range of FSHR density basally observed at the cell surface was 10-80 FSHR/μm^2^ across all experiments. For standardization of data analysis and to eliminate receptor density as a variable factor, cells with FSHR expression levels of ~10-40 FSHR/μm^2^ were selected for analysis, as this was the physiological receptor density range previously reported for the FSHR (30) and other native GPCRs (38). To prevent the overestimation of FSHR association, coordinates localized within 15 nm of 15 consecutive frames were filtered using an add-on algorithm JAVA program. To determine the number of associated FSHRs and type of associated forms, from the localized and filtered x-y coordinates, a PD-interpreter JAVA program was employed to perform Getis-Franklin neighborhood analysis with a search radius of 50 nm. Reconstructed heat maps representing the different numbers of associated FSHRs were produced as a result.

### GloSensor™ cAMP Assay

Post-transfection, cells were pre-equilibrated for 2 hours at 37°C in 88% CO_2_-independent media (18045, Gibco) supplemented with 10% FBS and 2% GloSensor™ cAMP reagent stock, as per manufacturer’s instructions. Following this, cells were treated for 30 minutes at 37°C with 0-100 ng/ml of eFSH, FSH21/18, FSH24 or dg-eLHt. Real-time cAMP florescence was measured using a multi-mode plate reader (PHERAstar^®^ *FS*, BMG Labtech) using the parameter of 100 flashes per well, with a cycle time of 36 seconds.

### Cre-Luciferase Assay

Post-transfection, cells were stimulated in serum-free DMEM supplemented and treated with 0-100 ng/ml eFSH, FSH21/18, FSH24 or dg-eLHt for 4-6 hours at 37°C. As an early-response gene, we expected this length of treatment to be sufficient for rapid gene expression induction in concordance with previous work (15). At the end of the incubation period, cells were lysed and treated with the Dual-Luciferase^®^ Reporter Assay System, as per manufacturer’s instructions. Lysate preps were measured for cre-luciferase and *R-*luciferase (for internal transfection control) luminescence levels using a multi-mode plate reader (PHERAstar^®^ *FS*, BMG Labtech).

### Statistical Analysis

For PD-PALM studies, to compare the effect of FSHR ligands on the percentage of total FSHR homomers, ordinary one-way ANOVA, followed by Tukey’s multiple comparisons test were conducted. To compare the effect of different FSHR ligands on the percentage of FSHR homomer subtypes, we performed multiple unpaired t-tests followed by Holm-Šídák’s multiple comparisons. For each experiment a total of 3 individual sections/well were imaged containing 3-4 cells. For each section typically 15 ROIs, within cell borders, were analyzed as previously described (34). For GloSensor™ assays, a baseline read of each well was performed for 10 read cycles prior to FSHR ligand treatment. The average baseline value of each cell was subtracted from its respective FSHR ligand-treatment, from the same wells. The mean cAMP response was plotted over 30 minutes and second order smoothened graph with 10 neighbors was performed. The area under the curve (AUC) measurements at 2-, 5- and 15-minutes were determined by measuring the total area from the number of peaks. All GloSensor™ data were represented as fold change/basal. Comparisons of the AUC between FSHR ligands were carried out using one-way ANOVA, followed by Tukey’s multiple comparisons test. Cre-luciferase luminescence readings were normalized to *R*-luciferase luminescence readings from the same well, to control for transfection efficiency. All data were represented as fold change/basal. Analysis of concentration-response curve were made using two-way ANOVA, followed by Dunnett’s multiple comparisons test. Comparisons between FSHR ligands at specific concentrations were performed using two-way ANOVA, followed by Dunnett’s multiple comparisons test. For PD-PALM, a minimum of 3 independent experiments were performed, for GloSensor™ and cre-luciferase assays, a minimum of 3 independent experiments in triplicate were conducted. All data presented represent the mean ± SEM. All statistical evaluations were performed using GraphPad Prism V9, and significance was determined as a probability value of *p*<0.05.

## Results

### FSH Ligands Differentially Modulate FSHR Monomer and Homomer Complex Formation in a Temporal and Concentration-Dependent Manner

To determine the effects of differentially glycosylated FSHR ligands on cell surface FSHR oligomerization, we utilized the previously described single molecule imaging technique, PD-PALM, which afforded imaging of individual FSHR molecules to <10nm resolution (34). Two concentrations of FSH glycoforms were utilized, based on previous reports showing differential cAMP production evoked by FSH21/18 and FSH24, with ~50% of maximal cAMP production at 30 ng/ml in the concentration ranges assessed, and low-level cAMP production at 1 ng/ml (15).

**Figure 1:**
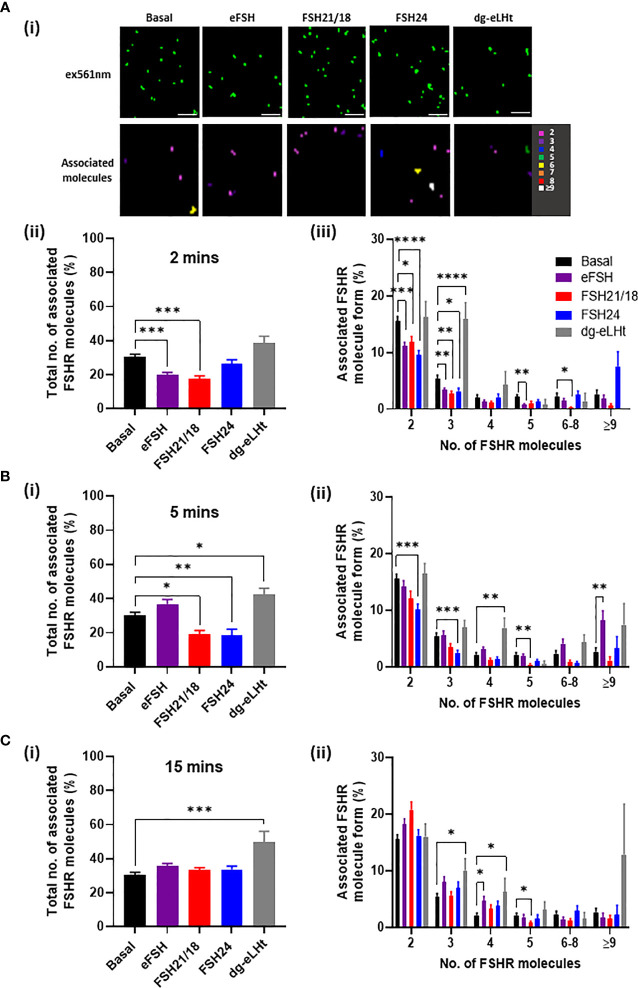
FSH ligands differentially modulate FSHR association. HEK293 cells transiently expressing HA-tagged FSHR were pre-incubated for 30 minutes with CAGE 552-HA antibody and treated with ± 30 ng/ml of eFSH, FSH21/18, FSH24 or dg-eLHt for either **(A)** 2 minutes, **(B)** 5 minutes or **(C)** 15 minutes, fixed for 30 minutes and imaged *via* PD-PALM. **(Ai)** Representative x-y coordinate plots of resolved FSHR molecules (upper panel) and reconstructed heat map of FSHR molecules following 2-minute treatment (lower panel). Images are 2µm^2^ from a 5µm^2^ area. *Scale bars*, 500nm. **(Aii)**, **(Bi)** and **(Ci)** Show the percentage of the total number of associated FSHR molecules; data analyzed using ordinary one-way ANOVA. **(Aiii), (Bii)** and **(Cii)** Shows the percentage of associated FSHR molecule form; 2 (dimer), 3 (trimer), 4 (tetramer), 5 (pentamer), 6-8, ≥9, with data analyzed using multiple unpaired t-tests. All data represent mean ± SEM of 3 independent experiments. *p < 0.05; **p < 0.01; ***p < 0.001; ****p < 0.0001.

HEK293 cells transiently expressing HA-FSHR were treated ± 30 ng/ml of FSH21/18 and FSH24 for 2-, 5- and 15 minutes. As a positive control, cells were also stimulated with the potent FSHR activator, eFSH, which is a naturally occurring analog of hypo-glycosylated FSH21/18. Conversely, as a negative control for cAMP activation, we utilized the FSHR β-arrestin biased agonist, dg-eLHt, which has been previously shown to display minimal cAMP production, with biased activation of β-arrestin (36, 37). Representative images (top panels) and heat maps depicting the number of associated molecules (bottom panels) were generated. Analysis of the basal number of associated FSHR showed that 30.2 ± 1.8% of FSHR were associated as dimers and oligomers (**Figure 1**), with ~70% as FSHR monomers. Analysis of the basal composition of associated cell surface FSHR showed 15.5 ± 0.8% resided as dimers and 5.5 ± 0.5% as trimers (**Figure 1**), suggesting that basally the majority of FSHR reside as lower-order homomers and monomers. Acute 2-minute treatment of HEK293 cells expressing FSHR with either eFSH or FSH21/18 significantly decreased the overall percentage of associated FSHR, with 20.0 ± 1.3% and 17.5 ± 1.6% associated as dimers and oligomers, respectively (**Figure 1Aii**). A decrease was observed in almost all FSHR homomeric subtypes (dimers, trimers, pentamers and 6-8 oligomers) (**Figure 1Aiii**). In contrast, treatment with FSH24 had no effect on the total percentage of associated FSHR, however modulation in the type of FSHR homomeric complexes was observed with a modest increase in ≥9 complexes and a decrease in dimers. Surprisingly, 2-minute treatment with dg-eLHt showed a trend for increasing FSHR association with 38.7 ± 3.8% of FSHR molecules associated (**Figure 1Aii**), with 15.9 ± 2.9% FSHR as trimers (**Figure 1Aiii**).

A 5-minute stimulation with eFSH treatment showed the percentage of FSHR associations to resemble basal (**Figure 1Bi**), suggesting a rapid re-organization of FSHR monomers into FSHR homomers. The documented fast binding kinetics of eFSH may explain this (17). FSH21/18, however, maintained a sustained reduction in FSHR homomers (**Figure 1Bi**). Interestingly, 5-minute treatment with FSH24 resulted in FSHR dissociation (**Figure 1Bi**), with a decrease in dimeric and trimeric FSHR homomers (*p*< 0.001) (**Figure 1Bii**). A 5-minute dg-eLHt treatment sustained the increase in FSHR association observed at 2 minute-treatment (**Figure 1Bi**), suggesting that different FSH ligands have distinct effects on FSHR oligomerization.

A more chronic 15-minute treatment with either eFSH, FSH21/18 or FSH24 resulted in FSHR total homomeric complex percentages resembling those of basal levels (**Figure 1Ci**). However, dg-eLHt-treated cells continued to show increased FSHR association (49.7 ± 6.4%) with increases observed in trimers, tetramers to ≥9 complexes (**Figure 1Cii**), further supporting the proposition that different FSH ligands can differentially modulate FSHR association.

**Figure 2:**
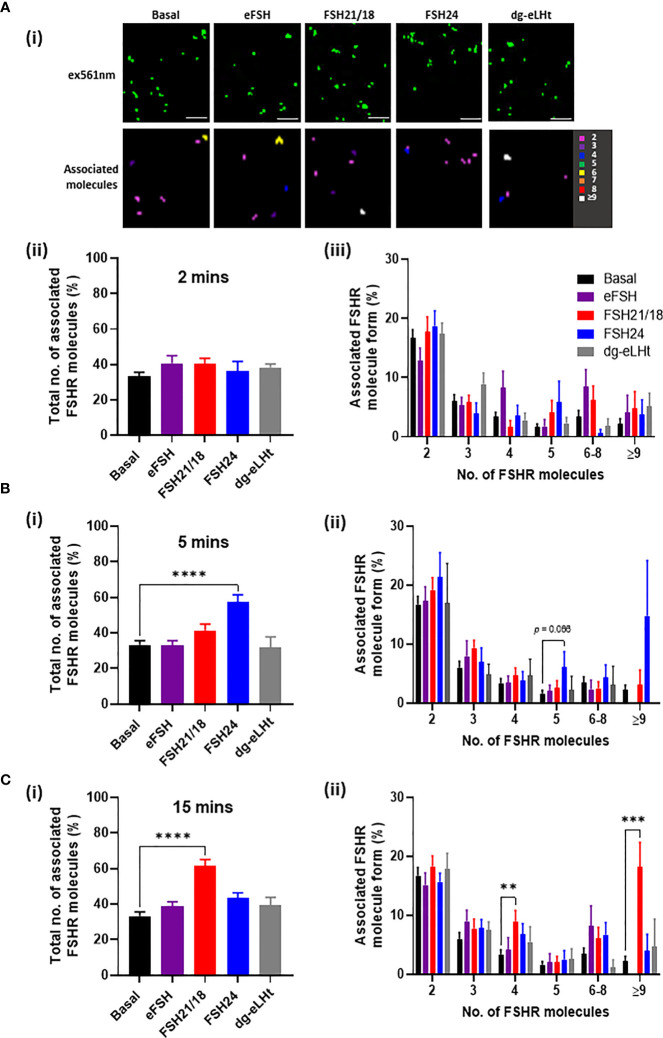
Low FSH ligand concentrations have distinct temporal effects on FSHR association. HEK293 cells expressing HA-tagged FSHR, labelled with CAGE 552-HA antibody and treated with ± 1 ng/ml of eFSH, FSH21/18, FSH24 or dg-eLHt for either **(A)** 2 minutes, **(B)** 5 minutes or **(C)** 15 minutes. Cells were fixed and imaged *via* PD-PALM. **(Ai)** Representative resolved localized FSHR molecules following 2-minute treatment (top panel) and heat map showing associated FSHR molecules). Images are 2µm^2^ from a 5µm^2^ area. *Scale bars*, 500nm. **(Aii)**, **(Bi)** and **(Ci)** The percentage of the total number of associated FSHR molecules; data analyzed using ordinary one-way ANOVA. **(Aiii), (Bii)** and **(Cii)** The percentage of associated FSHR molecule form; 2 (dimer), 3 (trimer), 4 (tetramer), 5 (pentamer), 6-8, ≥9; data analyzed using multiple unpaired t-tests. All data represent mean ± SEM of 3 independent experiments. *p < 0.05; **p < 0.01; ***p < 0.001; ****p < 0.0001.

Since FSH concentrations and glycosylation patterns are differentially regulated across the menstrual cycle (39) and have also been shown to change with age (13), we sought to determine the effects of FSH ligand concentration on FSHR association. As previously, HEK293 cells expressing FSHR were treated ± eFSH, FSH21/18, FSH24 or dg-eLHt for 2-, 5- or 15-minutes, but using 1 ng/ml of each. Representative reconstructed images of FSHR localizations and heat maps showing associated molecules following 2-minute treatment were generated (**Figure 2Ai**). Assessment of FSHR association following 2-minute treatment with all ligands revealed no significant changes in the total percentage of FSHR homomers (**Figure 2Aii**), nor the type of FSHR homomeric complexes observed (**Figure 2Aiii**), suggesting that lower concentrations of FSHR ligands had little effect on FSHR association at this acute time-point.

Similarly, 5-minute treatment with 1 ng/ml eFSH, FSH21/18 and dg-eLHt had no effect on FSHR association (**Figure 2Bi**). Interestingly, FSH24 induced a significant increase in FSHR association, with an increase in the formation of pentamers (6.1 ± 2.6%) (**Figure 2Bii**), contrasting to the dissociation of FSHR homomers observed with 30 ng/ml FSH24 shown above. Intriguingly, 15-minute treatment with FSH21/18 also induced FSHR association (**Figure 2Ci**), with an increase in FSHR tetramers (9.0 ± 1.8%) and ≥9 oligomers (18.2 ± 4.1%) (**Figure 2Cii**). FSH24-treated cells appeared to show FSHRs return to basal configuration (**Figure 2C**). Taken together, these data suggest that different FSHR ligands specify distinct re-organization of FSHR monomer, dimer, and oligomer populations in both a concentration- and time-dependent manner.

### FSHR Ligands Differentially Modulate cAMP Production

**Figure 3:**
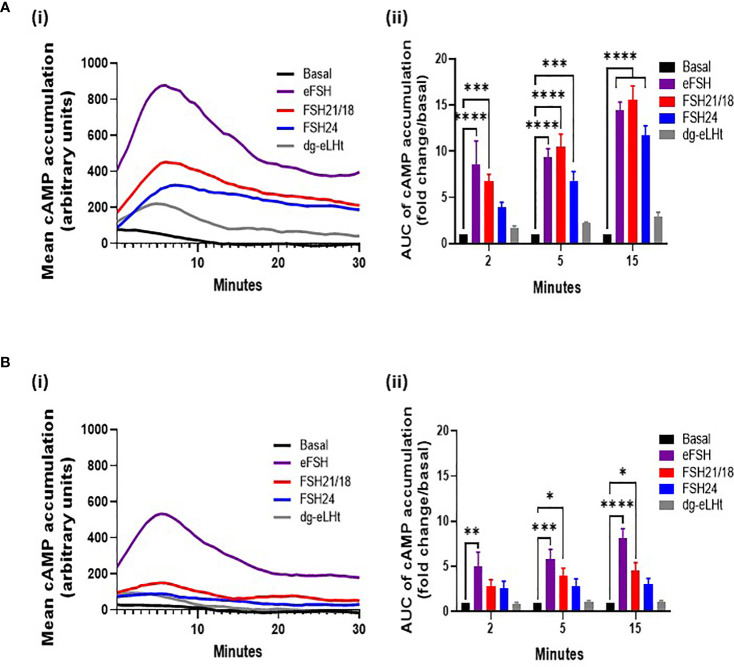
Different concentrations of FSH ligands differentially modulate cAMP production in a temporal manner. HEK293 cells expressing the HA-tagged FSHR and pGloSensor™-20F plasmid to assess live GloSensor™ cAMP kinetics. Cells were stimulated with either **(A)** ± 30 ng/ml or **(B)** ± 1 ng/ml of eFSH, FSH21/18, FSH24 or dg-eLHt. (i) Smoothened curve of the mean cAMP accumulation following treatment over 30 minutes (no error bars). (ii) AUC of the mean ligand-dependent cAMP accumulation at 2-, 5- and 15 minutes. AUC data was baseline subtracted and represented as fold change/basal. Data analyzed using two-way ANOVA. All data represent mean ± SEM of 3-5 independent experiments, measured in triplicate. *p < 0.05; **p < 0.01; ***p < 0.001; ****p < 0.0001.

Ligand-dependent modulation of the related luteinizing hormone receptor homomers and LHR/FSHR and FSH/GPER heteromers has been shown to regulate signal amplitude and specificity (32–34) To investigate if the time- and concentration-dependent changes in FSHR monomers and homomers observed at the plasma membrane correlated with modulation in cAMP signals in our cell system, we employed the cAMP GloSensor™ reporter, which afforded real-time monitoring of cAMP production. HEK293 cells expressing FSHR were treated ± 0-100 ng/ml of eFSH, FSH21/18, FSH24 or dg-eLHt. Full cAMP concentration-response curves showing the AUC and maximal response of cAMP accumulation were recorded (**Supplementary Figure S1**), and the 30- and 1 ng/ml data extrapolated for further analysis at the 2-, 5-, and 15-minute time points, for correlation with the time points utilized for PD-PALM experiments. The mean cAMP accumulated over 30 minutes following a 30 ng/ml treatment with all ligands were plotted (**Figure 3Ai**). A 2-minute treatment with either eFSH and FSH21/18 induced a significant increase in cAMP production of 8.6 ± 2.6- and 6.7 ± 0.8-fold change/basal, respectively (**Figure 3Aii**). There were no significant effects of either FSH24 or dg-eLHt, on cAMP production at this time point (**Figure 3Aii**). When compared and correlated with PD-PALM data, a trend was observed for 2-minute 30 ng/ml eFSH and FSH21/18 promoting dissociation of FSHR homomers into predominantly monomers (**Figures 1Aii, iii**), suggesting that dissociation of FSHR oligomers into monomers and re-organization of FSHR oligomeric complexes may, at least in part, promote acute cAMP production. Moreover, that no change or enhancement of FSHR oligomerization may facilitate low level production of cAMP.

Treatment for 5-minutes with FSH24 significantly increased cAMP (**Figure 3Aiii**). When compared to observations with PD-PALM data, a decrease in FSHR association at 5-minutes FSH24 treatment was observed (**Figure 1B**). This provided further support that FSHR dissociation into monomers may promote cAMP production. Differences in the magnitude of cAMP accumulation between eFSH, FSH21/18 and FSH24 were observed (**Supplementary Figure S1**) and may result from the temporal differences in the kinetics of FSHR homomeric complex dissociation and profile of FSHR homomers favored by different FSHR ligands. We observed predominantly eFSH- and FSH21/18-dependent pentamer dissociation with acute treatment (**Figures 1Aiii, Bii**) compared to FSH24-dependent FSHR dimer and trimer dissociation (**Figure 1Bii**). The dg-eLHt preparation failed to significantly stimulate cAMP production (**Figure 3Aii**), as compared to PD-PALM data, which showed increased FSHR oligomerization (**Figure 1**).

A 15-minute stimulation with either eFSH, FSH21/18 or FSH24 continued to significantly increase cAMP production (**Figure 3Aii**). However, from the maximal cAMP concentration-response curves (**Supplementary Figure S1Cii**), steady-state levels appeared to have been reached. Interestingly, at this time point, FSHR homomer arrangements predominantly resembled basal conditions in all treatment groups (**Figure 1C**), suggesting that this receptor configuration may be important in initiating FSHR signal activation, with other mechanisms such as receptor internalization important in maintaining cAMP production thereafter. As anticipated, dg-eLHt was unable to induce significant cAMP production at any time point analyzed (**Figure 3Aii**). PD-PALM data at the corresponding time point showing preferential re-arrangement of FSHR into higher order oligomers (**Figure 1**), suggesting that low level cAMP production (and potential β-arrestin recruitment and subsequent signaling) may be mediated, at least in part, by FSHR oligomer formation.

Next, we determined the effects of lower FSHR ligand concentrations that differentially modulated FSHR homomerization, on cAMP production. As with our previous PD-PALM experiments, we utilized 1 ng/ml of eFSH, FSH21/18, FSH24 or dg-eLHt and measured the mean cAMP accumulated over 30 minutes (**Figure 3Bi**). An acute, 2-minute treatment with all ligands, except eFSH, showed minimal increases in cAMP production in comparison to basal (**Figure 3Bii**). When compared to the PD-PALM data (**Figure 2**), these data correlated with a lack of effect on FSHR oligomerization at 2 minutes, following 1 ng/ml treatment with any FSHR ligand (**Figures 2Aii, iii**).

At 5- and 15 minutes, although we observed an eFSH-dependent increase in cAMP production of 5.8 ± 1.1- and 8.1 ± 1.1-fold, respectively (**Figure 3Bii**), when correlated to the PD-PALM data at these time points, no changes in the total percentage of FSHR homomers at the plasma membrane were observed (**Figure 2**). This suggests that there may be a dose-dependent threshold for different FSH glycosylated ligands to modulate FSHR homomerization. Small changes were observed in FSHR homomer subtypes, which may be important for modulating the magnitude of cAMP signaling, however this remains to be demonstrated. In contrast, FSH24 treatment at 5- and 15-minutes had no significant effect on cAMP production (**Figure 3Bii**). Interestingly, when correlating PD-PALM analysis, an increase in FSHR oligomerization was observed, predominately from enhanced formation of pentamers (**Figure 2Bii**), which may indicate that low level cAMP production may favor FSHR association. Supporting this observation, we observed increases in the total percentage of FSHR homomers with FSH21/18 treatment at 15 minutes (**Figure 2Ci**), correlating with low level cAMP production at the same time (**Figure 3Bii**). As anticipated, no significant changes in cAMP were observed following 2-, 5-, or 15-minute treatment with dg-eLHt (**Figure 3Bii**).

### FSHR Ligands Differentially Modulate Cre-Luciferase Activity

**Figure 4:**
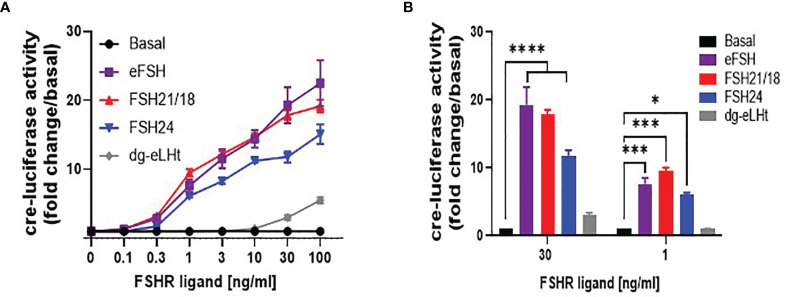
Differential FSH ligands stimulate cre-luciferase activity in a concentration-dependent manner. HEK293 cells were transiently expressing HA-tagged FSHR, cre-luciferase and *Renilla* luciferase plasmids. Cells were treated in serum-free media for 4-6 hours with increasing concentrations (0-100 ng/ml) of eFSH, FSH21/18, FSH24 or dg-eLHt. **(A)** concentration-dependent effects of FSH ligands on cre-luciferase activity, represented as fold change/basal. **(B)** Extrapolation of data from **(A)** to measure the effect of ± 30- and ± 1 ng/ml of eFSH, FSH21/18, FSH24 or dg-eLHt on cre-luciferase activity. Data represented as fold change/basal and analyzed using ordinary two-way ANOVA. All data points were normalized to the internal transfection efficiency control, *Renilla*-luciferase. Each data point represents mean ± SEM for 3-5 independent experiments, measured in triplicate. *p < 0.05; **p < 0.01; ***p < 0.001; ****p < 0.0001.

As the principal pathway activated by FSH/FSHR is Gαs/cAMP/PKA, which is physiologically important for regulating the expression of cre-response genes, including CYP19 essential for estradiol production (16, 19, 40–42), we went on to determine the effects of FSHR ligands on cre-luciferase reporter gene activity. HEK293 cells transiently expressing the HA-FSHR were co-transfected with cre-luciferase and *R*-luciferase (transfection efficiency control) and treated with ± 0-100 ng/ml of eFSH, FSH21/18, FSH24 and dg-eLHt for 4-6 hours. In line with the GloSensor™ data, eFSH and FSH21/18 were most potent at activating cre-luciferase for all concentrations ≥1 ng/ml when compared to basal in contrast to FSH24-treated cells (**Figure 4A**). As anticipated, dg-eLHt was unable to induce any changes in cre-luciferase activity at lower concentrations. However, at the higher concentrations of 30- and 100 ng/ml, dg-eLHt appeared to act as a weak agonist at the FSHR (**Figure 4A**), in corroboration with previous reports (36).

Comparison of 30 ng/ml treatments with FSHR ligands showed ligand-dependent differences in cre-luciferase activation, with eFSH and FSH21/18 significantly stimulating cre-luciferase activity by 19.3 ± 2.6- and 17.8 ± 0.7-fold increase over basal, respectively, in comparison to an 11.8 ± 0.8-fold increase for FSH24-treated cells (*p*<0.0001) (**Figure 4B**). This reflects similar differences observed between eFSH, FSH21/18 and FSH24 in 30 ng/ml GloSensor™ cAMP data (**Figure 3Aii**). Furthermore, when compared to FSH ligands eliciting changes in FSH homomerization, it suggests that the changes observed in FSHR complexes at the plasma membrane may contribute to modulating the magnitude of cre-responsive gene activation.

Comparison of cre-luciferase responses following 1 ng/ml treatment with eFSH, FSH21/18, FSH24 and dg-eLHt revealed both eFSH and FSH21/18 induced 7-fold increases in cre-luciferase activation (*p*<0.001) (**Figure 4B**). Additionally, FSH24 induced a comparable increase (6.1 ± 0.2-fold over basal) in cre-luciferase activity. This was interesting as differential regulation of FSHR homomeric forms and cAMP production was observed at this concentration. As predicted, dg-eLHt failed to significantly induce any increase in cre-luciferase activity (**Figure 4B**), further supporting its β-arrestin biased agonist activity.

## Discussion

**Figure 5:**
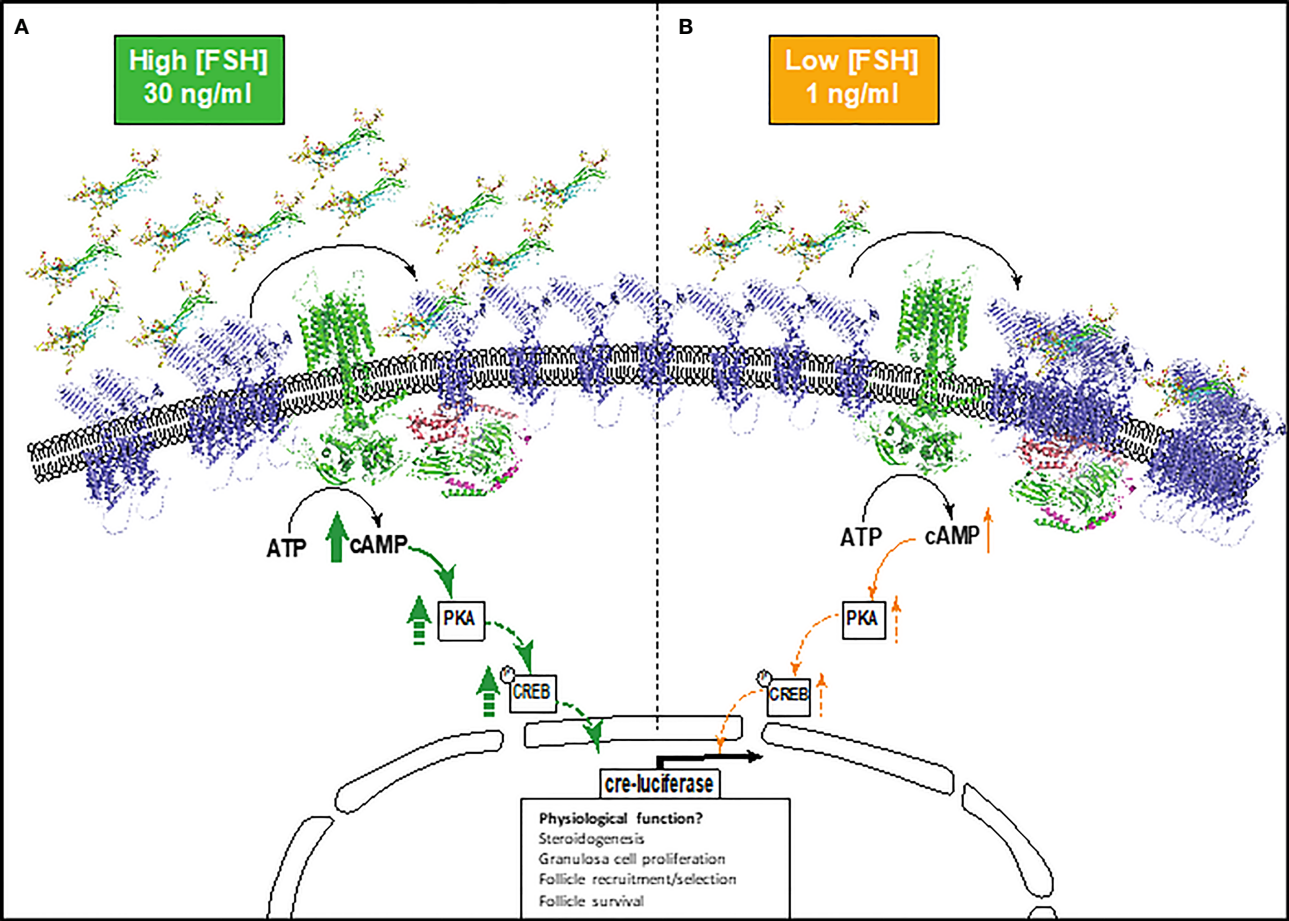
Schematic depicting the proposed model of FSHR monomer/oligomer-dependent modulation of cAMP signaling. **(A)** Higher physiological concentrations of FSH glycoforms (30 ng/ml), mediates the re-organization of FSHR oligomers into monomers at the plasma membrane, activating the Gαs-adenylyl cyclase-cAMP-PKA-CREB pathway and resulting in high cre-responsive gene activation. **(B)** Lower physiological concentrations of FSH glycoforms (1 ng/ml). FSHR monomer-homomer populations remain quiescent or further associate to promote FSHR oligomerization. This results in lower levels of cAMP and cre-responsive gene activation. This may have implications on granulosa cell functions including steroidogenesis, granulosa cell proliferation, follicle recruitment and selection, and follicle survival. Dashed arrows indicate proteins in the cAMP pathway that were not investigated, but are concordant with published literature. FSH glycoforms are modelled, FSHR is represented by an alpha-fold model (blue) and structural depiction of adenylyl cyclase (green).

FSH glycosylation variants have been previously shown to display differences in the magnitude and specificity of pathways activated (15, 17, 19, 43). Yet how FSHR decodes and propagates such signal diversity and differences in signal amplitude and duration remains unknown. GPCR homomerization is a well-recognized mechanism for modulating functional diversity and specifying signal responses (44, 45). Here we propose a mechanism for FSH glycoform-specific temporal and concentration-dependent regulation of FSHR homomerization, which correlates modulation of the amplitude and temporal activation of cAMP signaling (**Figure 5**).

We have shown that pituitary FSH glycoforms regulate FSHR homomerization in a time- and concentration-dependent manner. At higher physiological concentrations, eFSH and FSH21/18 rapidly dissociated FSHR homomers predominantly into monomers, correlating with significant increases in eFSH and FSH21/18-dependent cAMP production (**Figure 5A**). Interestingly FSH24 displayed slower temporal kinetics of modulating FSHR homomerization, but dissociated FSHR homomers into predominantly monomers at time points when cAMP production was significantly increased. These data are in concordance with early studies of the related glycoprotein hormone receptor, TSHR, where FRET and co-immunoprecipitation analysis revealed less active dimer and oligomer conformations dissociated into monomers upon TSH stimulation (46). Conversely, at high concentrations of the β-arrestin biased agonist, dg-eLHt, a rapid increase in FSHR homomerization was observed. For the purpose of this study, this FSHR biased agonist was used as a negative control for cAMP production, and this increase in FSHR oligomerization correlated with a limited ability to activate cAMP, consistent with previous reports (36). These data suggest that defined FSHR monomer, dimer and oligomeric forms may at least in part, modulate the magnitude of cAMP production. This proposition is interesting as FSH glycoforms have been demonstrated to be dynamically regulated during ageing, with modulation in the ratio of FSH21/18 and FSH24 and overall circulating levels of FSH as ovarian ageing proceeds (13), and reported changes in secretion across the menstrual cycle (13, 39). It is tempting to speculate that modulation of FSH glycoform and concentrations during these physiological processes may modulate FSHR homomer subtype and the dynamic shift between monomer-dimer-oligomer forms. This in turn may specify the amplitude and duration of cAMP signaling, with potential for signal specific physiological outcomes. Such FSH glycoform specific modulation of FSHR complexes and tempering of cAMP production may present ways for therapeutic exploitation in assisted reproduction. However, further research is required to understand the molecular detail and physiological control of this. These data also have implication for other GPCRs reported to homomerize/heteromerize, as ligand concentration is often overlooked and is particularly important for receptors with endogenous ligands that have diurnal/circadian/cyclical changes in secretion.

The glycoprotein hormone receptors have been previously reported to display inherent negative cooperativity, or functional asymmetry (26). This has been described for homomers for many GPCRs (47, 48) and has been proposed as a mechanism for mediating more graded responses. It has additionally been suggested that negative cooperativity may play an important role in many biological responses as it can cause marked threshold and ultra-sensitivity, allowing a biological system to filter out small stimuli and respond decisively to suprathreshold stimuli (48). Our observations of FSHR dissociation at high ligand concentrations and FSHR association/no change with low ligand concentrations support this idea, whereby FSHR oligomerization decodes the ligand threshold to regulate signal activation. In a physiological context within the ovary, such regulation may help prevent mass activation of FSHR, and fine-tune FSHR function during the fluctuations in FSH concentrations that are observed in different phases of folliculogenesis such as follicle recruitment, selection and ovulation (6, 49, 50).

Differences in binding affinity and the number of FSHR sites occupied by FSH21/18 and FSH24 have previously been reported, with FSH21 displaying a higher binding affinity to FSHR and occupying more FSHR (51, 52). Additionally, competition binding assays have shown that unlabeled eFSH and FSH21/18 was more efficacious at displacing ^125^I-FSH24 and ^125^I-FSH21/18 at lower concentrations than unlabeled hFSH24 (14), supporting the differences in FSHR binding affinities. In the context of this study, it is possible that these reported differences in the binding properties of the FSH glycoforms may have implications for the temporal differences observed in FSHR oligomer re-arrangement and dissociation into monomers observed with eFSH and FSH21/18 versus FSH24. However, future studies are required to determine how FSH glycoform-dependent differences in FSHR binding affinity and kinetics may drive changes in FSHR oligomerization.

We utilized the FSHR biased agonist, dg-eLHt, with known preferential β-arrestin signaling at lower concentrations (≤1nM) and weak cAMP activation (36), and observed concentration dependent modulation of FSHR oligomerization, with enhanced association at high concentrations. For other GPCRs, agonist dependent induction of homomerization has also been observed, including the dopamine D2 receptor homodimers (53). As dg-eLHt is a preferentially recruits β-arrestin, which has well established roles in receptor desensitization and internalization, we can’t rule out the induction of FSHR clustering, rather than FSHR oligomerization for initiation of FSHR internalization, particularly at high ligand concentrations. Indeed, interesting next steps will be to explore the effects of FSH glycoforms on the desensitization, internalization and trafficking of FSHR. It will be interesting to unpick how these observed differences in FSHR organization at the plasma membrane may direct FSHR internalization and trafficking, and understand the relationship between canonical Gαs coupling and β-arrestin recruitment and signaling. The association of FSHR and β-arrestin has long been established, with roles of this molecular scaffold in ERK phosphorylation (18, 54–56), with ligand activation rapidly phosphorylating a cluster of residues within the C terminus of FSHR to facilitate β-arrestin recruitment and receptor internalization (57). Interestingly, a recent study has suggesting FSH glycoform-specific differences in the dependency of β-arrestin for ERK activation (17). With the recently reported roles of ligand-dependent differences in regulatory ‘phosphorylation barcodes’ for other Class A GPCRs (58), it may be that FSH ligands generate differential phosphorylation barcodes resulting in ligand-specific modulation of FSHR trafficking and signal propagation. Recent reports have suggested that internalization of FSHR is required for initiation of FSH-dependent cAMP production (59), with low molecular weight FSHR agonists reported to differentially modulate FSHR exocytosis (59), which may explain differential profiles for activating cAMP. How FSH glycoforms direct FSHR internalization and trafficking, remains to be determined. However, the use of single molecule imaging and single particle tracking presents exciting opportunities to determine the spatial-temporal regulation of these processes and uncover how/if different FSHR complexes are routed through the endosomal machinery to modulate FSH ligand-dependent signaling.

FSH glycoforms have been demonstrated to activate additional non-canonical G protein-signaling including Gαq and Gαi linked pathways (56, 60, 61). It will be interesting to determine if FSHR oligomerization contributes to determining and specifying G protein-coupling within a FSHR oligomeric/monomer complex. Indeed, observations with LHR/FSHR heteromers support this idea, whereby an increase in heterotetramers was reported to drive the enhanced LH/LHR-dependent Gαq/11 signaling (32). Interestingly, the formation of FSHR and membrane bound estrogen receptors (GPER) heteromers (FSHR-GPER) have been recently reported (33), with a proposed role in reprogramming FSHR-related death signals into life signals, as a result of high density FSHRs (62) and/or too high cAMP (63). These data support the physiological roles of FSHR homomers and heteromers with potentially distinct biological functions. This may be important for extragonadal FSHR functions where FSH/FSHR has been shown to activate Gαi-dependent MEK/ERK NF-κB, and Akt signaling to enhance osteoclast formation (64–66) and Gαi-dependent induction of uncoupling protein-1 expression for FSH-dependent regulation of adipocytes (65, 67). However, this remains to be determined.

This study highlighted that ~70% of FSHR basally resided as monomers. This is in concordance with previous studies with the gonadotrophin hormone receptors, where the LHR was reported to reside as ~60% as monomers (34). Earlier studies into the Class A rhodopsin GPCR revealed that the majority of these receptors function as monomers despite their high concentrations within the plasma membrane (68). An important next step is to understand the role of FSHR monomers, and how they regulate the functionality and physiological responses of FSHR.

One of the limitations of this study is the utilization of HEK293 cells to study FSHR oligomerization, with transfected FSHR expression. A primary function of ovarian granulosa cells, in which FSHR are endogenously expressed, is to produce estrogen *via* aromatase expression. As a steroidogenic cell, the granulosa cell plasma membrane environment is cholesterol rich (69). The local membrane environment is increasingly recognized as an important factor regulating GPCR function (70–72) and GPCR homomer formation (73). To begin to understand the physiological context of this study findings, an important next step is to translate these findings into physiologically relevant cell types.

In conclusion, we have demonstrated that differential FSHR ligands modulate FSHR homomerization in a concentration and time-dependent manner. These data suggest that modulation of FSHR homomerization may provide a mechanism to fine-tune signal specificity and amplitude. This may be important means to decode the occurring cyclical and age-dependent changes in FSH concentration and glycosylation patterns in both a physiological and pathophysiological context. Moreover, modulation of FSHR homomerization may provide potential novel therapeutic avenues for targeting FSHR to improve IVF outcomes.

## Data Availability Statement

The original contributions presented in the study are included in the article/**Supplementary Material**. Further inquiries can be directed to the corresponding author.

## Author Contributions

KJ and GB conceived the study. KJ, GB, and AA supervised the study. UA, EC, and KJ carried out experiments. UA, EC, and KJ analyzed data. UA, KJ, AA, VB, and GB wrote and revised the manuscript. All authors contributed to the article and approved the submitted version.

## Funding

This work was supported by National Institutes of Health (P01AG029531), BBSRC grant (BB/R015961/1 and BB/R015961/2), and Society for Reproduction and Fertility Covid-19 support scheme for PhD Students 2021. 

## Conflict of Interest

The authors declare that the research was conducted in the absence of any commercial or financial relationships that could be construed as a potential conflict of interest.

## Publisher’s Note

All claims expressed in this article are solely those of the authors and do not necessarily represent those of their affiliated organizations, or those of the publisher, the editors and the reviewers. Any product that may be evaluated in this article, or claim that may be made by its manufacturer, is not guaranteed or endorsed by the publisher.
