# Supplementary material for: Differential FSH Glycosylation Modulates FSHR Oligomerization and Subsequent cAMP Signaling
Source: Front Endocrinol (Lausanne). 2021 Dec 3;12:765727. doi: 10.3389/fendo.2021.765727 (PMC8678890; doi:10.3389/fendo.2021.765727)
Supplement: Supplementary file 1 [file DataSheet_1.docx]

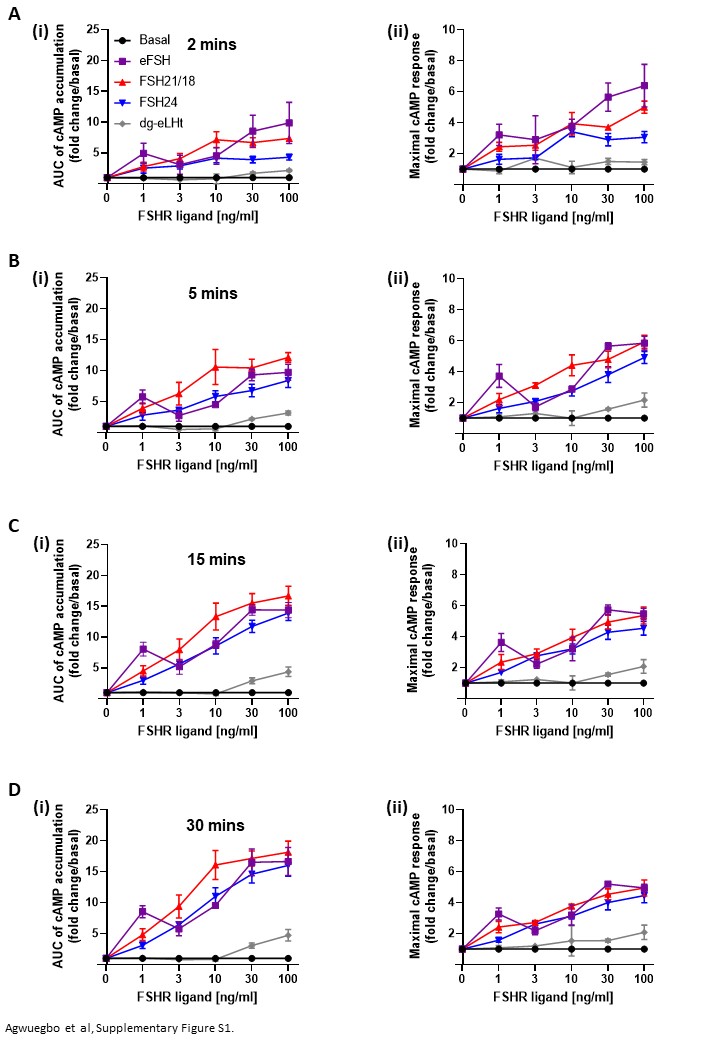


**Supplementary Figure S1. Concentration-dependent effects of FSH ligands on cAMP production.** HEK293 cells transiently co-expressing the HA-tagged FSHR and pGloSensor^TM^-20F plasmid were pre-equilibrated for 2 hours at 37°C and then treated for up to 30 minutes with increasing concentrations (0-100 ng/ml) of eFSH, FSH21, FSH24 or dg-eLHt. At **(A)** 2 minutes, **(B)** 5 minutes, **(C)** 15 minutes and **(D)** 30 minutes, measurements of the **(i)** AUC and **(ii)** maximal response were taken. Data represented as fold change/basal and analyzed using ordinary one-way ANOVA. All data represent mean ± SEM of 3-5 independent experiments conducted in triplicate. **p* < 0.05; ***p* < 0.01; ****p* < 0.001; *****p* < 0.0001.
